# A dataset of eco-evidence tools to inform early-stage environmental impact assessments of hydropower development

**DOI:** 10.1016/j.dib.2020.105629

**Published:** 2020-04-28

**Authors:** Ryan A. McManamay, Esther S. Parish, Christopher R. DeRolph

**Affiliations:** a Department of Environmental Science, Baylor University, Waco, TX 76798-7266, United States; b Environmental Sciences Division, Oak Ridge National Laboratory, Oak Ridge, TN 37831, United States

**Keywords:** Dams, River, Streams, Eco-evidence, Hydropower, Indicators, Environmental sustainability, Sustainability protocol

## Abstract

The datasets described herein provide the foundation for a decision support prototype (DSP) toolkit aimed at assisting stakeholders in determining evidence of which aspects of river ecosystems have been impacted by hydropower. The DSP toolkit and its application are presented and described in the article “Evidence-based indicator approach to guide preliminary environmental impact assessments of hydropower development” [1]. Development of the DSP and the output for decision support centralize around 42 river function indicators describing the dimensionality of river ecosystems through six main categories: biota and biodiversity, water quality, hydrology, geomorphology, land cover, and river connectivity. Three main tools are represented in the DSP: A science-based questionnaire (SBQ), an environmental envelope model (EEM), and a river function linkage assessment tool (RFLAT). The SBQ is a structured survey-style questionnaire whose objective is to provide evidence of which indicators have been impacted by hydropower. Based on a global literature review, 140 questions were developed from general hypotheses regarding the impacts of dams on rivers. The EEM is a model to predict the likelihood of hydropower impacting indicators based on a several variables. The intended use of the EEM is for situations of new hydropower development where results of the SBQ are incomplete or highly uncertain. The EEM was developed through the compilation of a dataset containing attributes of dams, reservoirs, and geospatial information on environmental concerns, which was combined with data on ecological indicators documented at those sites through literature review. The model operates through 247 “envelopes” and weighting factors, representing the individual effect of each variable on each indicator, all available through spreadsheets. Finally, the RFLAT is a tool to examine causal relationships amongst indicators. Inter-indicator relationships were hypothesized based on literature review and summarized into node and edge datasets to represent the structure of a graphical network. Bayes theorem was used estimate conditional probabilities of inter-indicator relationships based on the output of the SBQ. Nodes and edges were imported into R programming environment to visualize ecological indicator networks. The datasets can be expanded upon and enriched with more detailed questions for the SBQ, building upon the EEM with to develop more sophisticated models, and identifying new relationships for the RFALT. Additionally, once the tools are applied to numerous hydropower developments, the output of the tools (e.g. evidence of impacted indicators) becomes a very useful dataset for meta-analyses of hydropower impacts.

Specifications table

**Table:** 

Subject	Environmental Science
Specific subject area	Environmental impact assessment and environmental sustainability of river ecosystems in relation to hydropower development
Type of data	Tables
	Charts
	Graphs
	Microsoft Excel Macro-enabled workbooks (.xlsm)
	Source code for the R programming environment (.txt)
	Interactive visuals
	Tools and data files are described in Table 1.
How data were acquired	Global literature review on impacts of dams, questionnaire-survey development, geospatial analysis, and data summary analysis
Data format	Raw
	Analyzed
	Model parameters
	Tools for data summary and visualization
Parameters for data collection	Parameters varied by each data acquisition step: 1) Questionnaire was developed through an eco-evidence style literature review approach. At least 5 questions were required to address each of the 42 river function indicators. Each question required justification from at least 1 reference from the literature review. Questions were designed so that “yes” answers lead to more evidence of environmental impact. 2) For the predictive model, environmental variables were obtained from Parish et al. [3] and through spatial join procedures in ArcMap 10.2 software. 3) Hypothesized relationships among indicators were structured in a binary (0,1) matrix based on literature review. Hypothetically justified relationships were then empirically quantified through applications of the questionnaire to expert stakeholders. Graphical networks were constructed using the igraph package in the R programming environment.
Description of data collection	Data are associated with a toolkit. Tool development was guided by a systematic literature review of the impact of hydropower dams on river ecosystems, measured by 42 indicators. A survey-style questionnaire was developed to determine evidence of hydropower impact on each of the 42 indicators. Using geospatial analysis, a series of predictor variables were assembled to build a model predicting the impact of hydropower on indicators. Based on the literature review, causal relationships among indicators were established and visualized using graphical networks. All data and tools, including the questionnaire are available in the Mendeley Dataset cited below.
Data source location	Global distribution of dams, with emphasis on United States
Data accessibility	Repository name: Mendely
	Data identification number: 10.17632/dv3pz8xcsp
	Direct URL to data: http://dx.doi.org/10.17632/dv3pz8xcsp
Related research article	McManamay, R.A., E.S. Parish, C.R. DeRolph, A.M. Witt, W.L. Graf, A. Burtner, Evidence-based indicator approach to guide preliminary environmental impact assessments of hydropower development. Journal of Environmental Management. 265(2020): 110,489. 10.1016/j.jenvman.2020.110489

## Value of the data

• These data (and tools) provide evidence of which functional components of river ecosystems are most likely impacted by hydropower development and important to address within studies conducted in environmental impact assessments.
• Owners of hydropower facilities undergoing permitting or licensing, stakeholders involved in hydropower licensing procedures, and individuals responsible for environmental impact assessments of hydropower facilities can benefit from the data and apply the tools.
• Applications of the tools to numerous hydropower development case studies provides rich output data to support future meta-analyses of the impacts of hydropower on river ecosystems.
• By using these datasets in early stages of hydropower planning and licensing, knowledge gaps can be addressed more rapidly and lead to increased efficiency, thereby providing more resources for impact studies and mitigation designs.

## 1 Data description

The data described in this article are associated with three main tools that comprise a Decision Support Prototype (DSP) Toolkit aimed at assisting stakeholders in identifying the most likely aspects of river ecosystems impacted by hydropower development. The background and development of each tool was described in McManamay et al. [1]; however, each of the datasets behind the tools are described in this paper more fully. A series of 42 river function indicators [1,2] were used to characterize divergent aspects of river ecosystems while also serving to consolidate the dimensionality of these complex systems into a manageable number of measures. These indicators were developed from a comprehensive literature review of the environmental impacts of hydropower [3] and are associated with six main categories of impacts to river systems: biota and biodiversity, water quality, hydrology, geomorphology, land cover, and river connectivity. The toolkit and supporting data, including the questionnaire,. are provided in the Mendeley Dataset cited within the specifications table.

The three tools comprising the DSP toolkit and associated data include:

1) Science-Based Questionnaire (SBQ): A series of structured survey-style 140 questions for understanding impacts of dams on river ecosystems were developed through a global literature review. A spreadsheet program was developed to summarize the results of questions into evidence of dam impacts on the 42 ecological indicators. Output is provided in tabular and graphical/chart formats.
2) Environmental Envelope Model (EEM): The EEM is a model to predict the likelihood of hydropower impacting indicators based on a several variables. The intended use of the EEM is for situations of new hydropower development where results of the SBQ are incomplete or highly uncertain. A dataset containing attributes of dams, reservoirs, and geospatial information on environmental concerns were compiled and combined with data on ecological indicators measured at those sites. The data were used to develop models predicting impacts of dams on ecological indicators. A total of 247 envelopes and weighting factors, representing the individual effect of each variable on each ecological indicator, were developed in a spreadsheet program.
3) River Function Linkage Assessment Tool (RFLAT): The purpose of RFLAT is to examine causal relationships amongst indicators. Based on literature review, a node and edge dataset was developed representing causal relationships (“edges”) between ecological indicators (“nodes)”. Bayes theorem was used estimate conditional probabilities of inter-indicator relationships based on the output of the SBQ. Nodes and edges were imported into R programming environment to visualize ecological indicator networks.

Data and tools are provided by a series of four files, along with an instruction manual as a step-by-step guide in the use of tools (Table 1). Each tool may require more than one file to operate fully. The SBQ relies on files B and C (Table 1) and operates through Macro-enabled Microsoft Excel Worksheets that include several spreadsheets. Table 2 provides a list of spreadsheets within files B and C and their function. The spreadsheets include macro-enable features, such as navigation buttons and print commands. Files B and C are almost identical with the exception that file B is intended for evaluating existing hydropower facilities, whereas file C is intended for new hydropower development. The EEM is only available in file C; hence, it is intended for new hydropower development only.

**Table 1:** Files, their description, and the relevant tools they support provided within the Decision Support Prototype Toolkit.

File Code	File Name	Related Tools	Description
A	Instruction_Manual.pdf	NA	A guide with step-by-step instructions to use the tools
B	DST_EHA&NPD_RFLAT.xlsm	SBQ; RFLAT	Macro-enabled Microsoft Excel Worksheet pertaining to existing hydropower dams or theaddition of power to non-powered dams. The worksheet includes the SBQ tool and the node and edge datasets used within the RFLAT tool.
C	DST_ NSD_RFLAT.xlsm	SBQ, EEM, RFLAT	Macro-enabled Microsoft Excel Worksheet pertaining to new hydropower development. The worksheet includes the SBQ tool, the EEM model, and the node and edge datasets used within the RFLAT tool.
D	RFLAT_R_code.txt	RFLAT	Text file of R programming code used to generate network diagrams of relationships among river functions. Relies on the node and edge datasets generated in Files B and C.
E	node_coords.csv	RFLAT	Template of node coordinates to arrange river functions in a structured fashion in network diagrams.
F	Function_Envelopes.xls	EEM	Microsoft Excel Worksheet with raw data used to develop envelopes for EEM, percentiles supporting the envelopes, the envelopes, and suggested weighting factors

**Table 2:** 

Spreadsheet	Description
Instructions	General overview of the questionnaire and a brief version of the details provided in the instruction manual
Questionnaire	Main component of SBQ. List of structured questions organized into major themes and pull-down lists of alternative responses to each answer and whether the question is relevant to specific biological taxa
Summary	Table summarizing the responses to questions associated with each of the river functions. This tabular summary includes the responses according to different spatial scales and taxa.
Bar Plots	These three figures summarize the results of the tabular information from the “Summary” Spreadsheet
Model	EEM Model Platform. Predicting a range of likelihoods of river functions impacted by hydropower development based on several coarse attributes (File C only)
Spider Diagram	Diagram provides a different way to visualize information from the “Summary” spreadsheet. The spider diagram graphically depicts the evidence that a river function is affected by hydropower development or operations. Specifically, the diagram represents the proportion of questions answered “yes” pertaining to each of the river functions.
Question Details	List of questions and their attributes including references, spatial scale, and other information. These specific attributes include the following:
	Project type - type of hydropower projects of potential relevance (EHA & NPD - existing hydropower assets and non-powered dams; All - refers to any type of hydropower project)
	Area - Spatial scale of relevance to a given question
	Biota - an indication of whether question is directly related to biota ("Y"= Yes, "N"=No).
	Taxa - an indication of whether answer to the question could be taxa-specific
	KeyQ - an indication of whether the question is a "key" structural question or not (where some answers might depend on others) ("Y"= Yes, "N"=No).
	Reference - literature reference used to develop the question
Node_temp	Template for nodes used to develop network diagrams within the igraph library (R programming). Nodes represent river functions whose frequency of question answers are automatically populated based on the results of the Questionnaire. The node template can be automatically exported to a .csv file. (RFLAT Tool)
Edge_temp	Template for edges used to develop network diagrams within the igraph library (R programming). Edges represent relationships among river functions. The strength of relationships are dependent upon results of the questionnaire, which are automatically populated. The edge template can be automatically exported to a .csv file. (RFLAT Tool)
Bibliography	Bibliography of all references used to create questions and indicator relationships
Q_DB	Database of unique question-river function combinations used to automatically calculate summary tables based on responses in the questionnaire. [Note: Alteration of the database content or structure will influence the summary output and diagrams. Users should not modify unless they have good reason to do so and are familiar with Microsoft Excel Visual Basic programming].
List	Used to create standard values for entry in Questionnaire. [Note: Alteration of the list will influence the questionnaire, summary output, and diagrams. Users should not modify unless they have good reason to do so and are familiar with Microsoft Excel Visual Basic programming].
Model_calc	Spreadsheet supporting calculations for the EEM Model. Contains all river function envelopes. (File C only)

The RFLAT relies on files B, C, D, and E. The node and edge datasets within files B and C are automatically populated with output from the SBQ and are used to generate graphical networks of relationships among indicators. File D is code written in for the R programming environment that imports .csv files automatically exported from files B and C. File E a .csv file of coordinates used for attractively structuring river functions in a hierarchical manner within graphical networks. We expand on each of the tools in the following sections.

### 1.1 Science-based questionnaire (SBQ)

**Fig. 1:**
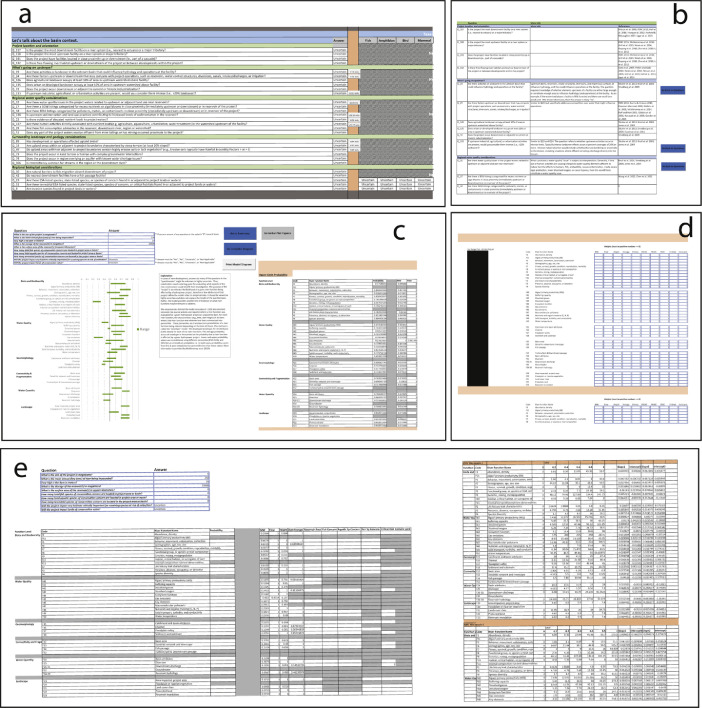
Spreadsheets within the Microsoft Excel macro-enabled program (Files B and C). (a) “Questionnaire” spreadsheet with blue box highlighting one of the macro buttons to navigate to (b) the “More_Info” spreadsheet providing more justification and background literature, (c) the “Model” spreadsheet where attributes of a hydropower development and associated environmental concerns can yield likelihood of impacts to river function indicators, (d) user-specified weighting factors within “Model” spreadsheet that influence relative importance of variables in the model, (e) “Model_calc” spreadsheet showing likelihood estimation panel and one part of the envelopes supporting the calculations.

The SBQ operates through the spreadsheets outlined in Files B and C (Table 1). The SBQ principally operates through the Questionnaire, a list of 140 questions (Fig. 1a). Questions are considered "generic" to identify common environmental effects of hydropower and dams on river functions. All questions must be answered “yes”, “no”, “uncertain”, or "not applicable". Furthermore, to affirmatively answer a question as “yes” or “no”, some form of evidence must be provided (e.g., data, analysis, picture, literature, website, stream gage reading, etc.). All questions are structured in such a way that “yes” answers lead towards more evidence for a given river function being affected by the facility. However, questions answered "uncertain" also provide evidence of river functions where more information or data is needed. If questions cannot be answered “yes” or “no” confidently and with evidence, questions should be answered as “uncertain” or “not applicable”. Some questions include phrases such as “significant” or “large” and require interpretation by the user. In these cases, a macro-enabled button is provided to navigated users to a separate “More Info” spreadsheet to provide more background information behind each question and literature references (Fig. 1b).

In some cases, questions may not be relevant to the specific context, such as particular types of hydropower development. For instance, environmental assessments evaluating the addition of hydropower to existing non-powered dams typically only consider the environmental effects of adding electrical generation infrastructure (e.g., turbines, penstocks, powerhouse) and not the a priori effects of the dam and reservoir. In these cases, answers to questions targeting dam development, in general, might be deemed “not applicable”.

The questions (and associated river functions) are also organized by spatial scale (Basin, Project, Reservoir, and Downstream) and according to which types of taxa (e.g., fish, amphibian, bird, etc.) may be relevant. The spatial scale is automatically built into the tool, but users can specify which taxa are relevant to a given question by answering "yes" under each taxa column.

**Fig. 2:**
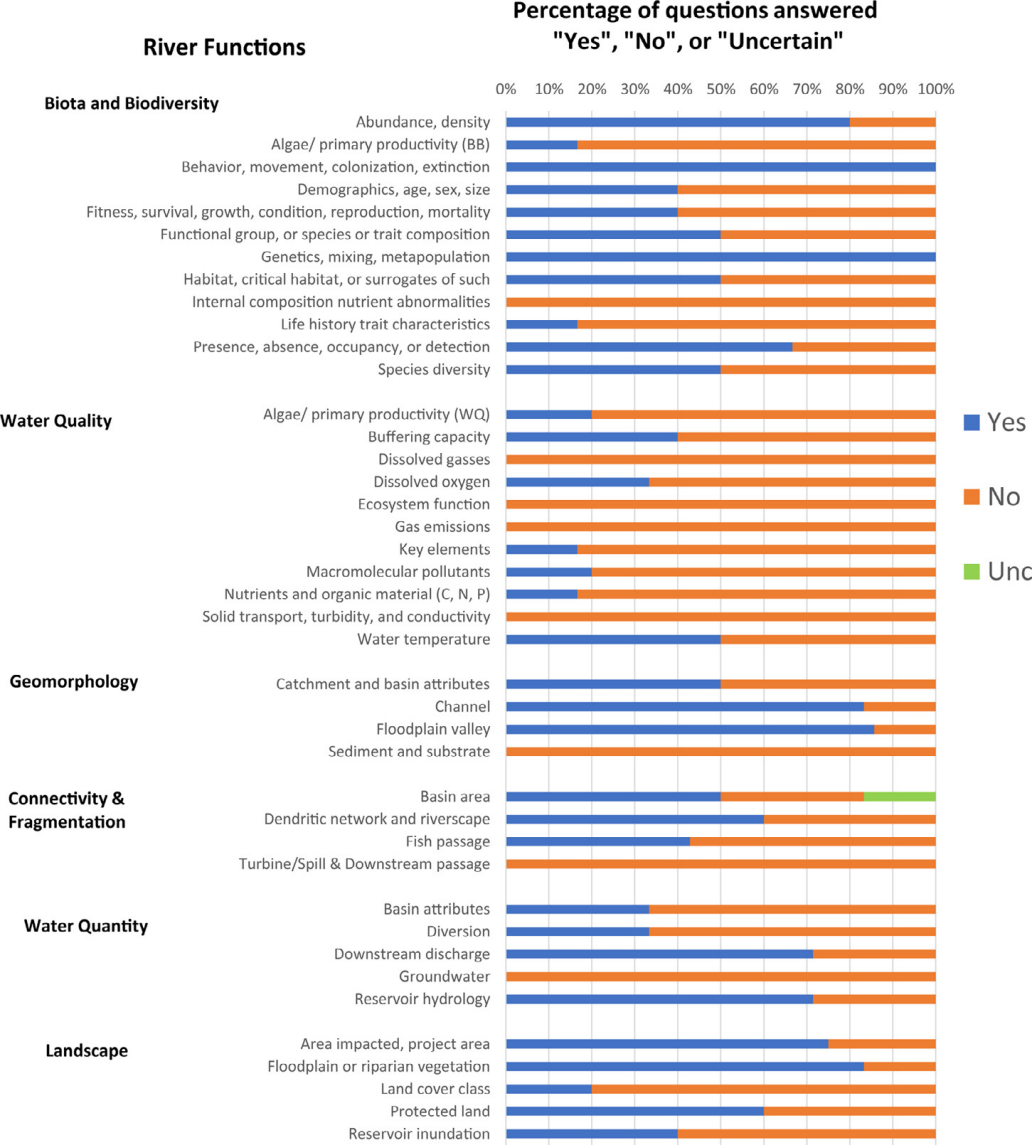
An example of the bar plot output based on outcomes of the Questionnaire. The percentage of questions answered “yes”, “no”, or “uncertain” for each river function indicator are summarized.

**Fig. 3:**
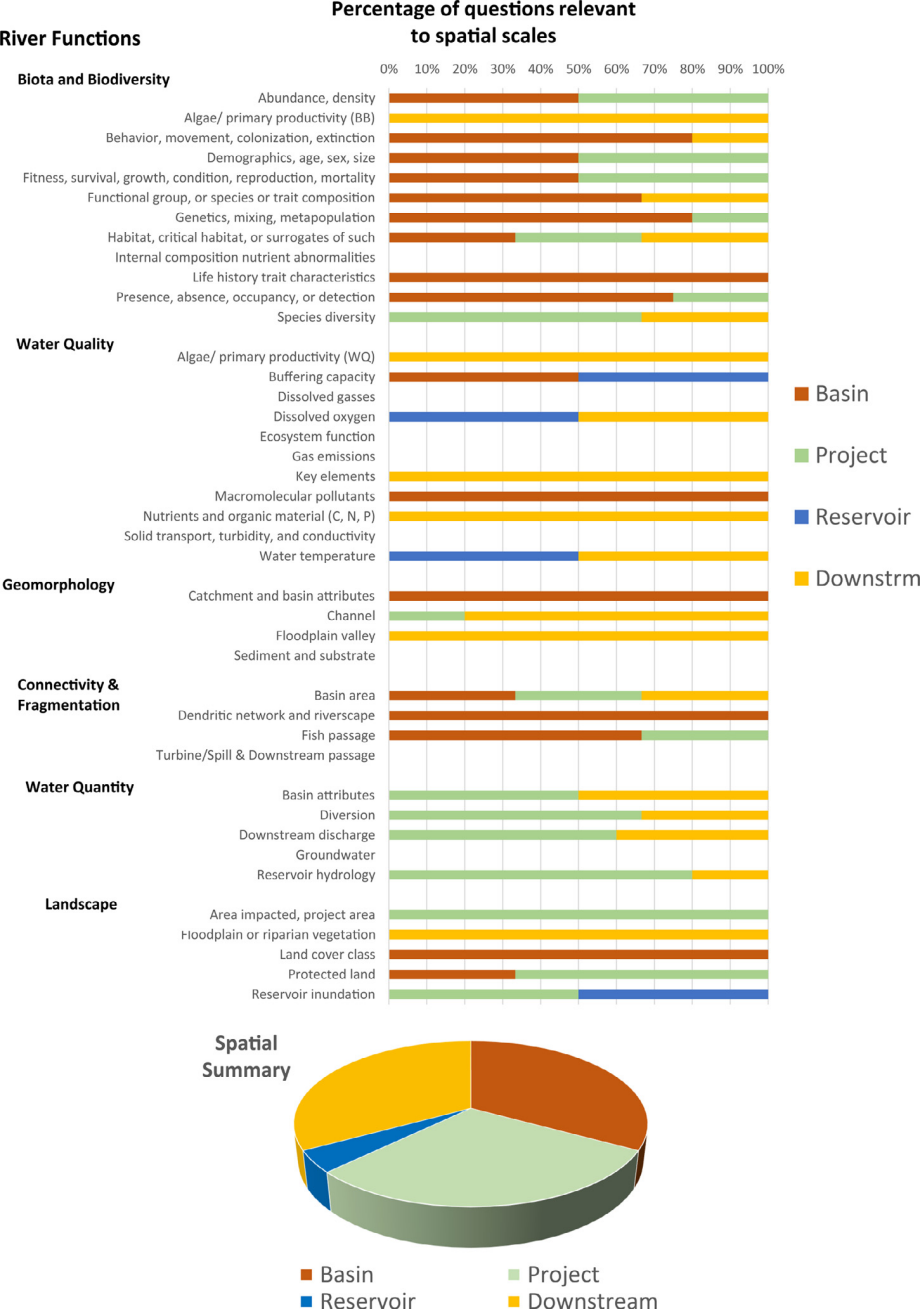
An example of the bar plot output summarizing questions answered “yes” from the Questionnaire and their associated spatial scale.

**Fig. 4:**
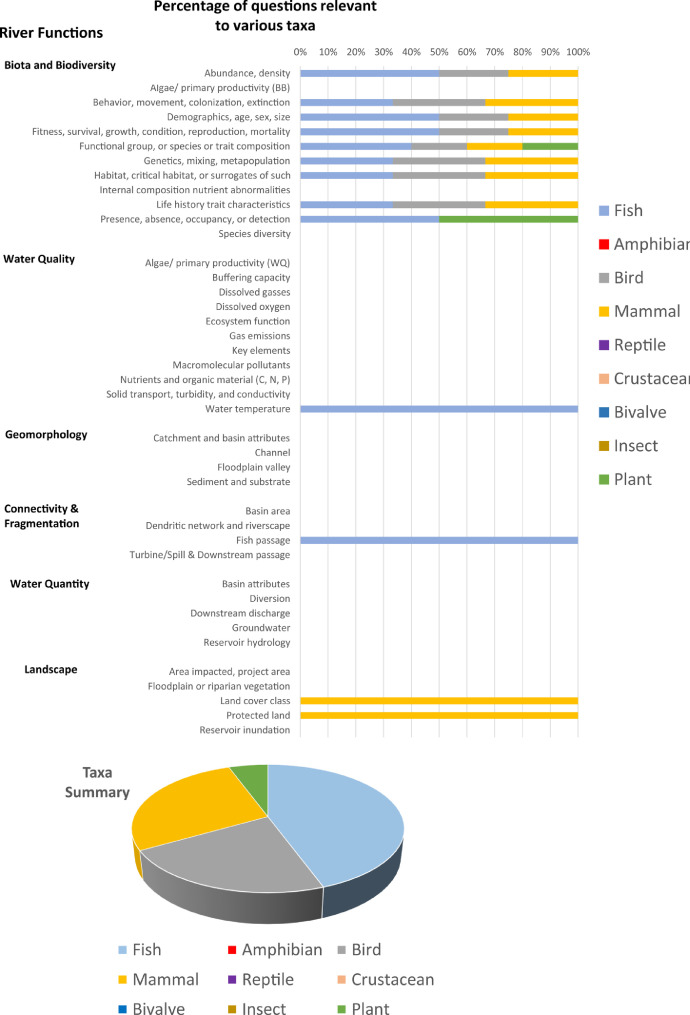
An example of the bar plot output summarizing questions answered “yes” from the Questionnaire and their associated relevance to different biological taxa.

**Fig. 5:**
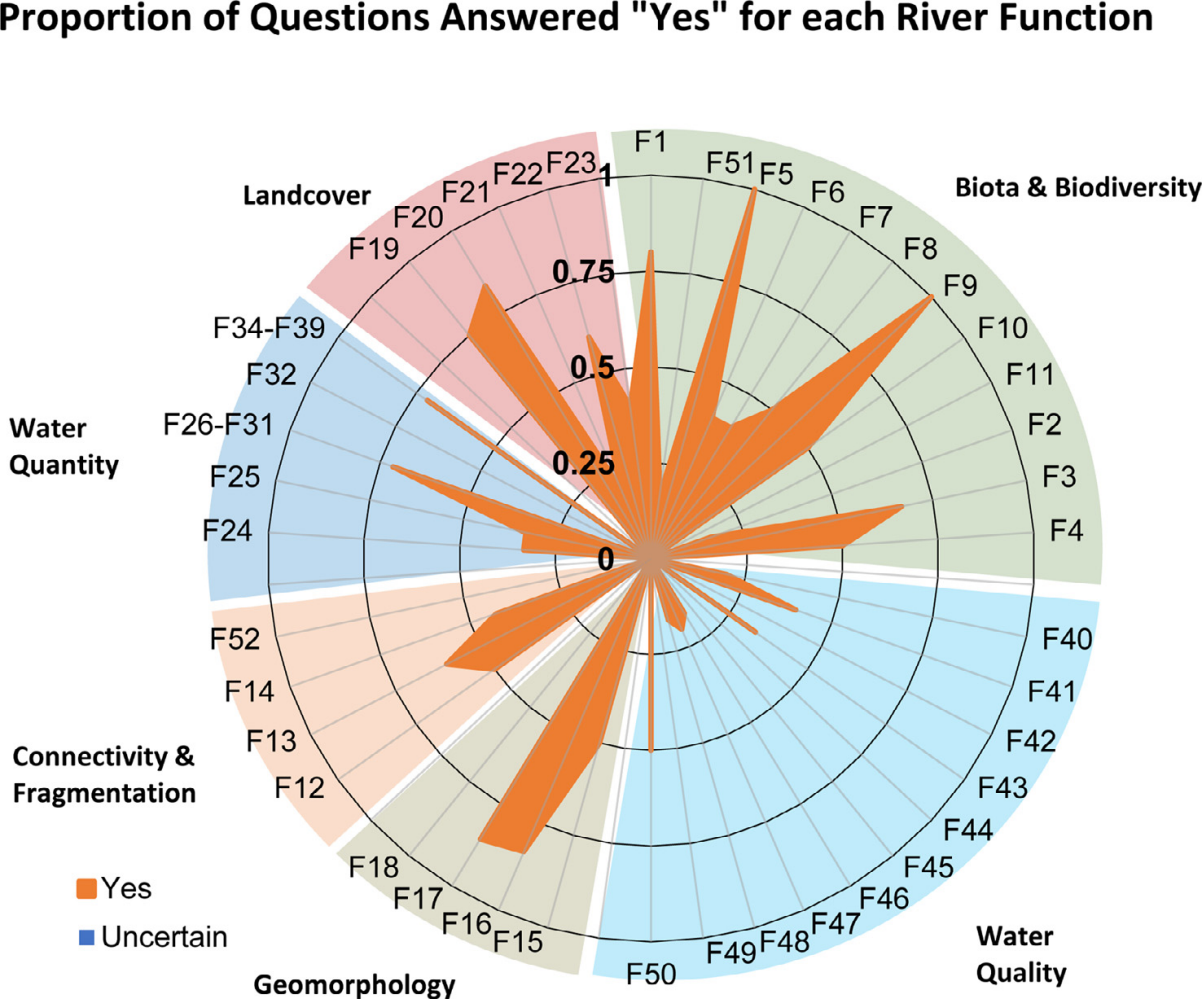
Example of a spider diagram output, which uses the same information captured in Fig. 2, but summarizes the information in a different way.

Each question pertains to at least one river function but may pertain to multiple river functions. On average, there are 5 questions supporting each river function, but numbers of questions per function may range from 4 to 11. Based on all answers, the total “yes”, “no”, and "uncertain" responses for a given river function are totalled, and provided in tabular form (“Summary” spreadsheet) or graphical form (“Bar_Plots”, and “Spider Diagram” spreadsheets) (Fig. 2, Fig. 3, Fig. 4, Fig. 5). An internal database (“Q_DB”) keeps track of all responses to all responses, spatial scale relevance, taxa-relevance, and the river functions applicable to all responses. Summaries of “yes”, “no”, or “uncertain” responses to all river function indicators are provided in tabular and graphical form for users to evaluate evidence (from 0 to 1) for any river function (Fig. 2, Fig. 3, Fig. 4, Fig. 5).

### 1.2 Environmental envelope model (EEM)

In cases of new development, answers to many of the questions in the SBQ might be unknown or highly uncertain. Thus, stakeholders need a starting point for evaluating what aspects of the river environment could benefit from investigation. The purpose of the EEM is to estimate the likelihood of a given river function being affected by a hydropower project based on a few attributes of that project. While the model relies on empirical data, it should be viewed as highly uncertain and does not replace the results of the questionnaire. Rather, the model provides another line of evidence of what river functions may be relevant to address. The EEM is a predictive model of environmental impacts of hydropower suggested for use in situations of new development, i.e. File C listed in Table 1.

All envelopes and VBA programming required to estimate likelihoods are provided in File C. The raw data used to develop envelopes is provided in File F and includes a list of river function indicators documented for a given dam and attributes for each dam used as predictor variables in the EEM.

On the “Model” spreadsheet within File C, users can fill out values within the table at the top of the page using the best available data (Fig. 1c). Missing values are allowed. Users have the option of selecting weights to influence the importance of each predictor variable (Fig. 1d), although calibrated weights are provided and are recommended for further use (also provided in File F). All calculations for envelopes are made within the “Model_calc” spreadsheet in file C (Fig. 1d). This spreadsheet provides the probability values for all 247 envelopes (Fig. 1d). Each envelope represents the individual influence of each predictor variable on the likelihood of impacting a river function indicator. Users are cautioned to not adjust any values or formatting on this spreadsheet unless they are familiar with the program and underlying data.

**Fig. 6:**
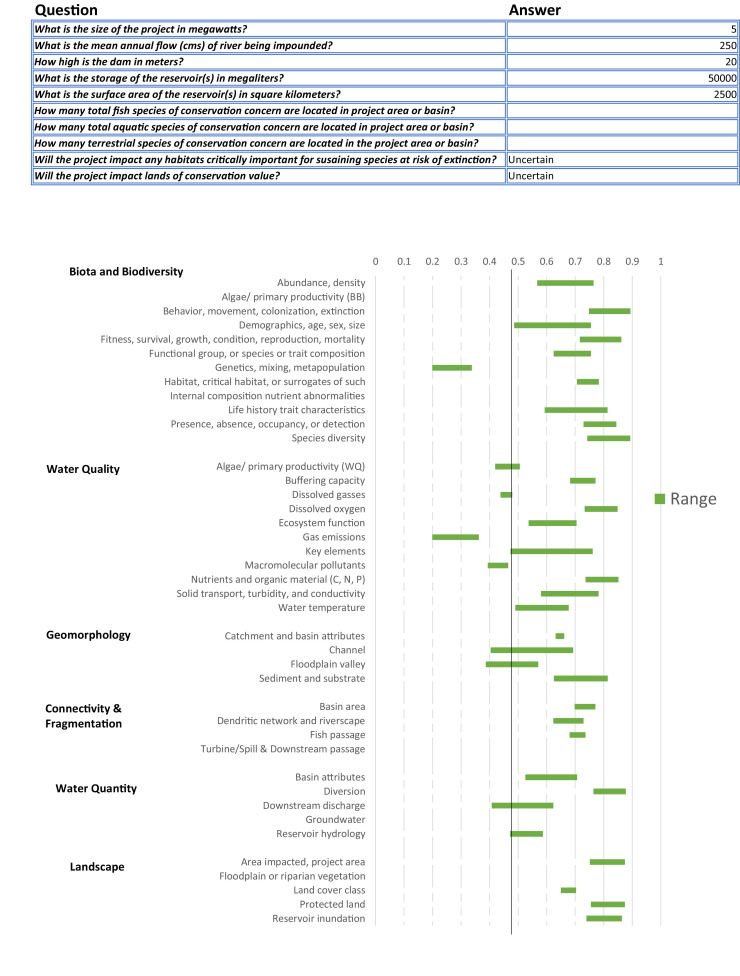
Example of the range plot output from the Environmental Envelope Model showing the range of likelihoods that a river function indicator is impacted. The values entered to render those likelihoods are also provided in the table above the range plot.

**Fig. 7:**
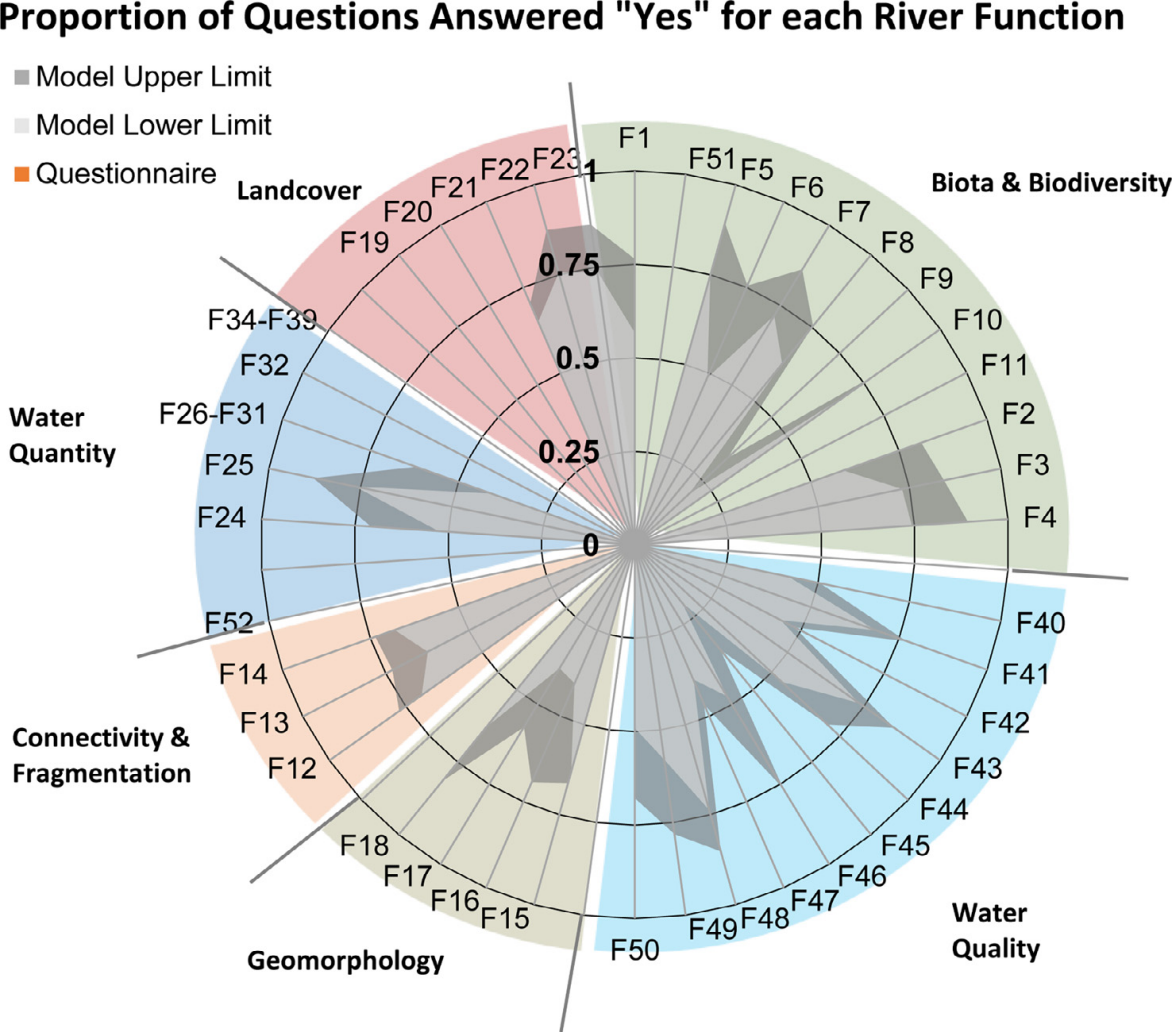
Example of the spider diagram output showing results of the range plot from the Environmental Envelope Model (same information from Fig. 6).

Probability values for both upper and lower thresholds are provided and can be interpreted as maximum and minimum values, respectively. Probability values from envelopes for all predictor variables are combined to calculate a range of likelihood of river function indicator impact (see methods section). Once the table is filled, the thresholds will automatically populate with values, which are reflected in the range plot (Fig. 6). The upper and lower thresholds are also reflected in the Spider Diagram and are plotted along with results of the SBQ (Fig. 7).

### 1.3 River function linkage assessment tool (RFLAT)

The RFLAT is used to examine linkages among river functions. It is dependent upon the results of the SBQ. The RFLAT requires that users be familiar with R programming environment. It operates under the assumption that the strength of relationships among river functions are dependent upon the combined strength of evidence of impacted individual river functions based on the results of the SBQ. However, this could yield a high number of non-sensical relationships; therefore, any relationship must be first supported through a hypothetical justification. Pairwise directional relationships between all indicators were generated with binary indications of justified (1) or non-justified (0) causal relations.

**Fig. 8:**
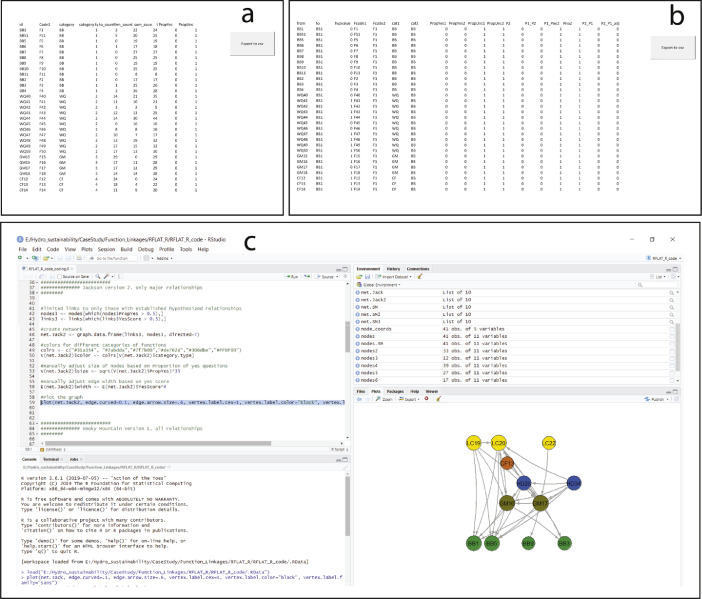
Data and programs used for the River Function Linkage Assessment Tool (RFLAT). (a) the Node dataset used to represent indicators as nodes in graphical networks, (b) the Edge dataset used to represent relationships among nodes, and (c) the R programming environment (R studio) with an example of generating a graphical network representing inter-indicator relationships. Note that the node and edge dataset spreadsheets are provisioned with an export macro-button to save each file as a .csv. Both files are updated with information from the Questionnaire.

The RFLAT relies on files B, C, D, and, optionally, file E listed in Table 1. Within files B and C, the Node_template and Edge_template spreadsheets are automatically populated with results from the SBQ (i.e., the questionnaire) (Table 2). The Node_template (Fig. 8a) is s a list of river function indicators and several attributes, such as the frequency of total relationships with other indicators or the proportion of questions answered “yes” for that indicator. Variable descriptions for the Node_template are provided in Table 3. The Edge_template (Fig. 8b) represents relationships among river function indicators. Variable descriptions for the Edge_template are provided in Table 4. Relationships are dependent upon the presence of a justified relationship and the conditional probability that indicator x (*I_x_*) has an influence on indication y (*I_y_*). McManamay et al. [1] presents and explains the following equation using Bayes theorem to calculate the conditional probability or strength of relationship of *I_x_* on *I_y_*:

(1) $$p\left(I_{y}|I_{x}\right)=\;H\left(\frac{p\left(I_{x}|I_{y}\right)p\left(I_{y}\right)}{p\left(I_{x}|I_{y}\right)p\left(I_{y}\right)+p(I_{x}|∼I_{y})p\left(∼I_{y}\right)}\right)$$

**Table 3:** Variables and their descriptions for the Node_template spreadsheet.

Variable	Description
Id	River function indicator identifier
Code1	Alternate code for each indicator
Category	Main category for each indicator useful for sub-selections
category.type	Numeric code for each category useful for sorting
to_count	Number of dependent relationships from other river function indicators
frm_count	Number of causal relationships to other river function indicators
sum_count	Sum of to_count and frm_count
PropYes	Proportion of questions answered "yes" related to the river function indicator
PropUnc	Proportion of questions answered "uncertain" related to the river function indicator

**Table 4:** Variables and their descriptions for the Edge_template spreadsheet.

Variable	Description
From	Id of River function indicator serving as origin of edge relationship
To	Id of River function indicator serving as recipient of causal relationship
Hypvalue	*H* or Binary indication of hypothetical relationship where "0″ represents an unjustified (or nonsensical) relationship and "1″ indicates a relationship is justified by literature review and a valid hypothesis
Fcode1	Alternate code for "from" id
Fcode2	Alternate code for "to" id
cat1	Main category for the "from" river function indicator
cat2	Main category for the "to" river function indicator
PropYes1	Proportion of questions answered "yes" for the "from" river function indicator
PropYes2	Proportion of questions answered "yes" for the "to" river function indicator
PropUnc1	Proportion of questions answered "uncertain" for the "from" river function indicator
PropUnc2	Proportion of questions answered "uncertain" for the "to" river function indicator
P2	*p*(*I_y_*) or the proportion of questions answered yes for Indicator y
P1_P2	*p*(*I_x_*|*I_y_*) or the probability of questions answered yes for Indicator x, given *p*(*I_y_*)
P1_Pno2	*p*(*I_x_*| ∼ *I_y_*) the probability of questions answered yes for Indicator x, given indicator y is not impacted by hydropower
Pno2	*p*( ∼ *I_y_*) the probability Indicator y is not impacted by hydropower
P2_P1_adj	*p*(*I_y_*|*I_x_*) – the conditional probability that Indicator y is influenced by Indicator x adjusted by the hypothetical value, H

**Fig. 9:**
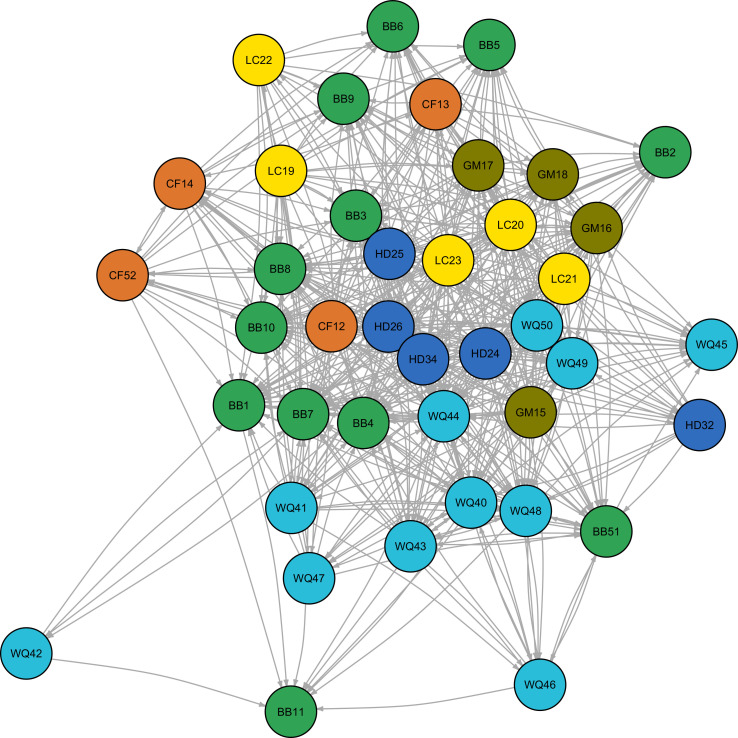
Example of an unstructured graphical network developed from the River Function Linkage Assessment Tool (RFLAT) representing indicator relationships for a hydropower development.

**Fig. 10:**
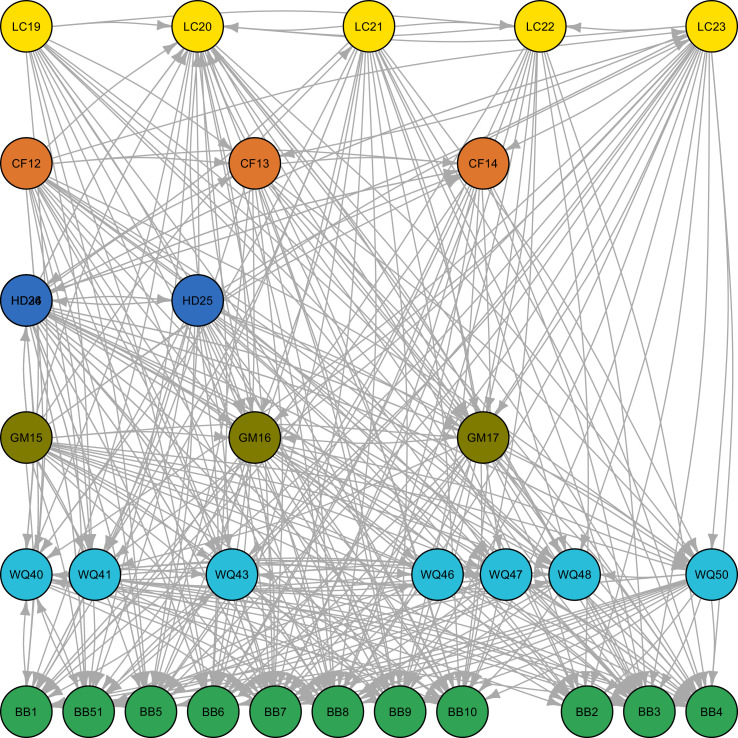
Example of an structured graphical network developed from the River Function Linkage Assessment Tool (RFLAT) representing indicator relationships for a hydropower development. Structured graphical networks are developed using coordinates from the “node_coords.csv” file.

Once the SBQ is completed, the Node_template and Edge_template spreadsheets can be automatically exported to .csv files using the macro buttons (Fig. 8a–b). These .csv files are then imported in the R programming environment (Fig. 8c). Programming code is provided in file D, which should be copied and pasted into the R interface. The code provided is reliant on the igraph package in R [4]. Igraph provides alternative styles of plotting network diagrams based on relationships. File E listed in Table 1 is a series of coordinates for river function indicators to provide a standardized structure for plotting nodes. This is optional if users desire. Fig. 9, Fig. 10 provide examples of alternative diagrams of the same network based on plotting functions.

## 2 Experimental design, materials, and methods

Methods are described in McManamay et al. [1]. Therefore, we provide a brief overview of methods while elaborating on specific areas not provided by McManamay et al. [1]. In particular, we elaborate on details of the EEM.

### 2.1 Science-based questionnaire (SBQ)

Questions supporting the SBQ were determined through scientific literature review and outcomes of consensus among multiple sources (See "Bibliography" in File B and C). Based on consensus of trends in literature, we developed general hypotheses regarding how dams influence river environments specific to each river function indicator. While it is recognized that dams and specific rivers are complex and inherently context-specific, there is much scientific literature that suggests that some environmental responses are generic to dams, given certain properties of the structure and the river. Questions were structured such that “yes” answers lead to more evidence of hydropower impacting a river function indicator.

### 2.2 Environmental envelope model (EEM)

The basis of the EEM model is an extension of the envelope method developed for species distribution modeling when only presence of occurrence is available [5,6]. An envelope refers to a curve representing optimal habitat conditions for a species within a range of values for a given environmental variable. Based on locations of where an organism is found, envelopes are calculated using percentiles of environmental values at those locations. Percentiles are then converted into probabilities of habitat suitability. Envelopes for species habitat suitability typically assume a trapezoid shape, where 10th and 95th percentile values of environmental variables translate into optimal habitat suitability (probability=1) and values lower or higher than the minima and maxima, respectively, are assigned probability values of 0.

**Fig. 11:**
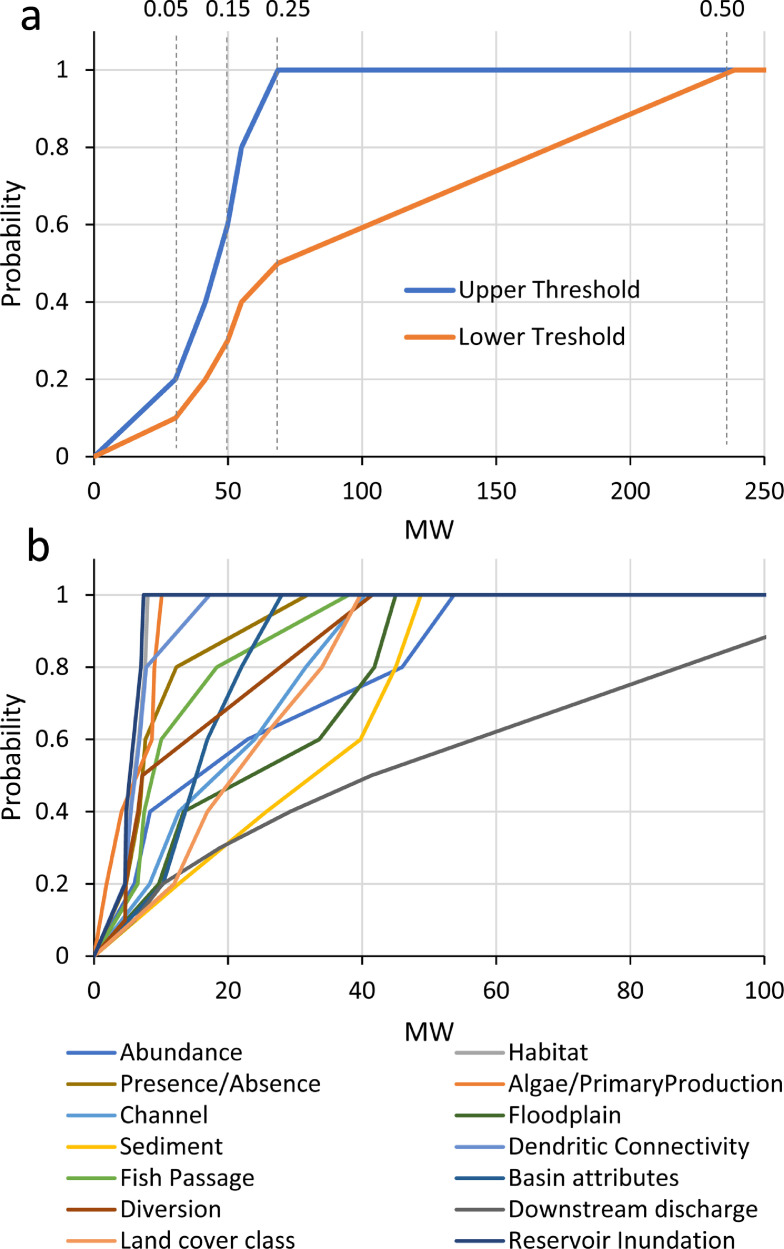
Example of river function indicator envelopes for supporting the Environmental Envelope Model. (a) An example of upper and lower threshold envelopes predicting the probability of impact of project generating capacity (MW) on the Water Temperature river function indicator (F50). Dashed lines and numbers represent percentiles of MW values associated with probabilities. (b) Example of the upper thresholds for envelopes predicting the probability of impact of project generating capacity (MW) on 14 different river function indicators.

We extended the concept of habitat envelopes to that of river function indicators, where the likelihood of impacts to river function indicators can be predicted based on attributes of the dam and environmental variables. However, envelopes predicting river function “suitability” do not approximate trapezoidal shapes, but rather curves of increasing probabilities of impact with higher values for dam attributes (e.g. dam height) or environmental concerns (e.g., species of concern). An example of river function envelopes is provided in Fig. 11. Predictor variables used to develop envelopes are described in Table 5.

**Table 5:** Predictor variables used in developing the Environmental Envelope Model (EEM), their description, and source.

Variable	Description	Source
MW	Generating capacity in megawatts as an indication of the size of the facility	Parish et al. [3]
Height_m	Height of the dam in meters	Parish et al. [3]
Flow_cms	Discharge of dam m^3^ s^−1^	Parish et al. [3]
Stor_ml	Storage of the reservoir in megaliters	National Anthropogenic Barrier Dataset [7]; HydroSource Existing Hydropower Assets [8]
SurfaceArea	Surface area (km^2^) of the reservoir	National Anthropogenic Barrier Dataset [7]; HydroSource Existing Hydropower Assets [8]
FSOC	Number of fish species of concern occurring in watershed containing hydropower project	NatureServe [9]
TASOC	Total number of aquatic species of concern occurring in watershed containing hydropower project	NatureServe [10]
TSOC	Number of terrestrial species of concern occurring in watershed containing hydropower project	NatureServe [10]
CritHab	Spatial intersection of federally engangered or threatened species’ critical habitats with project reservoir or proximate downstream environment	ECOS [11]
ConsLand	Spatial intersection of protected lands with project reservoir or proximate downstream environment	Protected Areas Database-US [12]

The empirical data behind the model consisted of 1380 documented instances in the global literature where a river function was evaluated for impact at a given hydropower project or unpowered dam [3]. Literature included peer-reviewed journal articles, Federal Energy Regulatory Commission license orders and environmental impact assessments, and sustainability protocol documents from the International Hydropower Association and the Low Impact Hydropower Institute [3]. For all 1380 instances, three variables were available from Parish et al. [3]: facility generating capacity (megawatts), river discharge, and height of the dam. However, the other 7 variables mentioned earlier were only available for dams within the United States, which consisted of 793 instances of river functions documented at hydropower facilities. Sources of data used for predictor variables are listed in Table 5. We created two datasets to support the development of river function envelopes.

For each river function, predictor variables (e.g., MW, dam height) were summarized into percentiles based only on locations where that river function was documented. Percentiles were then translated into probabilities (*p*) of a river function being impacted depending on the dam attribute – this is the essence of an envelope (Fig. 11). We created lower and upper threshold envelopes for each predictor variable and river function indicator. The lower threshold envelope was created where predictor values >= 50th percentile equals the maximum *p* = 1, and where percentiles of 0.1, 0.2, 0.25, 0.3, and 0.4 equal *p* values of 0.2, 0.4, 0.5, 0.6 and 0.8 (Fig. 11a). The upper threshold was based on predicted values >= 25th percentile equal to *p* = 1 and percentiles of 0.05, 0.1, 0.15, and 0.20 equal *p* values of 0.2, 0.4, 0.6, and 0.8, respectively (Fig. 11a). We then developed a non-linear function by decomposing each envelope into four slopes based on four portions in percentiles: 1) 0 > *p* < 0.4, 2) 0.4 >= *p* <=0.6, 3) 0.6 > *p* < 1, and 4) *p* = 1. Examples of envelopes representing the individual influence of MW on 14 river functions is provided in Fig. 11b.

As described by McManamay et al. [1], probabilities (*p*) predicted by envelopes for all predictor variables for each river function indicator are combined into a suitability score (SS) using the following Equation:

(2) $$SS_{f}=\frac{\sum_{i}^{n}p_{i}w_{i}}{\sum_{i}^{n}w_{i}}$$

**Fig. 12:**
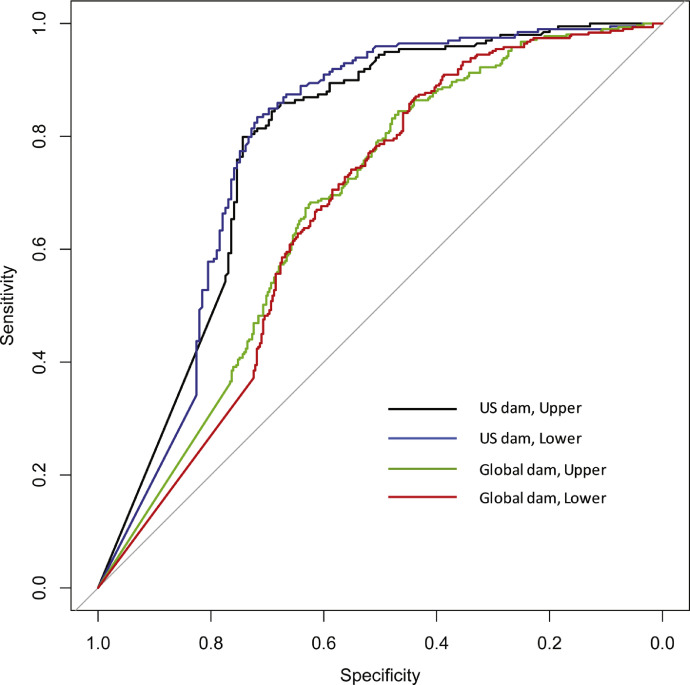
Receiver-Operating-Curves (ROC) for four different Environmental Envelope Models (EEM). The Global Dam model represented more data but only relied on MW, dam height, and river discharge as predictor variables. The US Dam model was constructed using less observations but included all predictor variables represented in Table 5. Upper and lower refer to upper and lower threshold EEMs.

Where *w* is a weighting factor for the *i*th predictor variable for each river function. Non-linear optimization was used to parameterize *w* values [1]. Area-Under-the-Curve (AUC) values for Receiver Operating Curves (ROC) were used to evaluate the performance of EEM models relative to observed presence–absences in river function indicators for a subset of the literature data where river function absences could be inferred [1]. Out of the 1380 global instances where river functions were documented and three predictor variables were available, the subset of presence–absence data comprised 744 observations (Global-dam), whereas for the 793 instances documented only in the US where all predictor variables were available, the subset resulted in 395 observations of presence–absence data (US-dam). These two datasets, in conjunction with upper and lower threshold envelopes, led to four different EEM models and associated evaluations of performance for each. For the Global-dam dataset, the upper and lower threshold EEM models both had AUC values of 0.67. For the US-dam dataset, the upper and lower threshold EEM models had AUC values of 0.78 and 0.79, respectively. ROC curves are provided in Fig. 12.

### 2.3 River function linkage assessment tool (RFLAT)

The structure behind the RFLAT is the development of node and edge datasets, which were created to be compatible with the igraph package in the R programming environment. Node datasets were essentially a list of all river function indicator IDs whereas edge datasets were lists of pairwise indicator combinations organized in such a way that causal indicator IDs were listed under the “from” column, whereas dependent indicator IDs were listed under the “to” column. Although all pairwise indicator combinations are listed in the edge dataset, those hypothesized as having a valid causal relationship, based on literature review, were flagged using a binary value of 1 or 0 indicating justified or unjustified by hypotheses, respectively. The frequencies of each indicator exerting causal influence or serving as the recipient of influence by another indicator were summarized as the number of “from” and “to” relationships supported by hypotheses for each node.

Variables within node and edge datasets are also automatically populated by the results of the SBQ. The proportions of questions answered “yes” or “uncertain” for each river function indicators are transferred to each node within the node and edge dataset. In the case of the edge dataset, there are two sets of values for the proportion of questions answered “yes” and “uncertain” relating to both indicators participating in a relationship. As described in McManamay et al. [1], the strength of relationships between river function indicators is calculated as a conditional probability using Bayesian theorem outlined in Eq. (1). These probabilities can be used as thresholds to influence the size and complexity of graphs of inter-indicator relationships.
